# Impact of transfer from non-acute care centers on clinical outcomes in patients with congestive heart failure

**DOI:** 10.1016/j.ahjo.2023.100251

**Published:** 2023-01-17

**Authors:** Ahmad Gill, Omar Al-Taweel, Emily He, Yousif Al-Baghdadi, Mohammad Jaradat, Luay Alalawi, Jibran Rana, Chowdhury Ahsan

**Affiliations:** a Department of Internal Medicine, University of Nevada, Las Vegas, Las Vegas, NV, United States of America; b Department of Cardiology, University of Nevada, Las Vegas, Las Vegas, NV, United States of America; c Department of Internal Medicine, Loma Linda University, Loma Linda, CA, United States of America; d Department of Cardiology, Lenox Hill Hospital, New York City, NY, United States of America; e Department of Cardiology, Harbor-UCLA Medical Center, Torrance, CA, United States of America

**Keywords:** Congestive heart failure, Non-acute care, Transfers, Nursing homes, Urgent care centers

## Abstract

**Study objective:**

To compare the clinical outcomes in patients with congestive heart failure who are transferred to an acute care hospital from non-acute care centers with patients who are admitted as regular hospital admissions.

**Design:**

This was a retrospective cohort study.

**Setting:**

We utilized the National Inpatient Sample database from 2016 to 2018.

**Participants:**

Our cohort consisted of hospitalized patients who were at least 18 years old with a primary diagnosis of congestive heart failure.

**Interventions:**

These patients were either transferred from non-acute centers or presented as regular hospital admissions.

**Main outcome measurements:**

We matched patients in a greedy nearest neighbor 1:1 model with caliper set at 0.2. Multivariable logistic regression, adjusted for age, sex, race and comorbidities, was used to compare mortality in our matched cohort.

**Results:**

This study included 35,010 non-acute care transfers and 951,189 regularly admitted patients. Compared to patients who were not transferred, non-acute care transfers were older, predominantly female, White and less racially diverse. After matching, there were 6689 patients in each cohort. When adjusted for age, race, sex and comorbidities, non-acute care transfers with congestive heart failure had 2.20 times higher odds of suffering in-hospital mortality compared to regular, non-transferred admissions (aOR 2.20, 95 % CI: 1.85–2.61; *p* < 0.001).

**Conclusion:**

Our findings illustrate that non-acute care transfers are a vulnerable population that require additional medical support in the acute care setting.

## Highlights

• Non-acute care transfers were older, predominantly female and White.
• Non-acute care transfers had 2.20 times higher odds of in-hospital mortality.
• Every race had higher rates of mortality in the non-acute care transfer cohort.

## 1 Introduction

Non-acute care is an umbrella term that encompasses numerous subsets of healthcare. It includes assisted-living centers, intermediate care facilities and outpatient physician clinics [1], [2]. Non-acute care facilities are popular across the United States for numerous reasons. Nursing homes and assisted living centers help support our older population [3], [4]. Intermediate care centers, such as urgent care clinics, are becoming more prevalent due to ease of access and increasing healthcare demand [1], [5], [6]. Non-acute care facilities help treat patients that do not require intensive medical management. In theory, this allows for a more focused distribution of hospital resources towards acutely ill patients. This decreases emergency department burden and also reduces financial costs for both our patients and healthcare system [7], [8]. As the number of non-acute care centers continues to expand to meet the increasing healthcare demand, it is imperative that we understand these long-term ramifications on our healthcare system [3], [9]. Only a few studies have compared patients who are transferred to an acute care hospital from non-acute care centers with patients who are admitted as regular hospital admissions. A prior study by Elkaryoni et al. suggests that patients with an ST-elevation myocardial infarction (STEMI) who are transferred from a non-acute care center have higher odds of in-hospital mortality than patients who are non-transferred STEMI admissions [2]. To our knowledge, no study yet exists that conducts a retrospective analysis on the outcomes of non-acute care transfers with congestive heart failure (CHF). Thus, in this study, we aim to explore the short-term prognostic outcomes of patients who are transferred to acute care hospitals from non-acute care centers with congestive heart failure.

## 2 Methods

This was a retrospective cohort study comparing adult patients who are transferred to the hospital from non-acute centers with patients who are non-transferred hospital admissions between 2016 and 2018. Patients were selected from the National Inpatient Sample (NIS) database, which is the largest publicly available all-payer medical sample in the United States. The NIS provides a 20 % probability sample of non-federal hospital discharges. All discharges from these hospitals are recorded and weighted to ensure that they are representative of the national demographic [10]. Each identified discharge record includes one primary diagnosis and up to 29 secondary diagnoses, using the International Classification of Diseases, Tenth Edition, Clinical Modification (ICD-10-CM). We chose 2016 as the beginning of the study period as this was the first full calendar year for ICD-10-CM code usage.

Our cohort consisted of hospitalized patients 18 years or older. We identified patients who were either transferred from non-acute centers or not transferred at all using the NIS data element “TRAN_IN.” Using ICD-10-CM codes, we then identified hospitalizations with a primary diagnosis of congestive heart failure. The specific ICD-10-CM codes that were included are listed in Appendix A. The primary outcome was the in-hospital mortality rate among congestive heart failure patients transferred from non-acute care centers compared to patients admitted as regular hospital admissions. The secondary outcomes included mortality by race, length of stay and total cost to the hospital.

In our unmatched cohort, regular Student's *t*-test was performed to compare normally distributed continuous variables, while Pearson χ^2^ tests were used to compare categorical variables. We estimated the propensity score by utilizing a logistic regression model based on patient's demographics (age, sex, health insurance, race), comorbidity variables (coronary artery disease, chronic kidney disease, chronic obstructive pulmonary disease, hypertension, obesity, prior myocardial infarction, type 2 diabetes mellitus) and hospital factors (bed size, location, region). We assessed for covariate balance using *t-*test and standardized differences. A standardized mean difference (SMD) of <0.1 was acceptable. We then matched non-acute care transfers with regular hospital admissions in a greedy nearest neighbor 1:1 model with caliper set at 0.2. In our matched cohort, paired *t*-test was used for continuous variables, while Pearson χ^2^ test was used for binary outcome variables.

Multivariable logistic regression, adjusted for age, sex, race and comorbidities, was used to compare mortality in our matched cohort. The comorbidities adjusted for included coronary artery disease, chronic kidney disease, chronic obstructive pulmonary disease, hypertension, obesity, prior myocardial infarction and type 2 diabetes mellitus. This was performed to eliminate confounding effects. The final effect size is reported as an odds ratio (OR) for binary variables and median with interquartile range (IQR) for continuous variables. The analysis was two-tailed with the threshold for significance set at *p* < 0.05. All analyses were performed using STATA Version 17.

## 3 Results

Among the 986,199 patients who met our inclusion criteria, 35,010 (3.5 %) were transferred from non-acute care centers (Table 1). In our unmatched cohort, the non-acute care transfers were older (77.2 years vs 71.2 years, *p* < 0.001), predominantly female (53.3 % vs 47.1 %, *p* < 0.001) and White (80.7 % vs 69.0 %, p < 0.001, Table 1). Non-acute care transfers had more patients in the highest APR-DRG Mortality score category (27.7 % vs 16.5 %, p < 0.001). The APR-DRG Mortality score is calculated from discharge billing codes and based on discharge diagnosis, pre-existing medical conditions and age. Non-acute care transfers were also more likely to have Medicare (83.0 % vs 72.2 %, *p* < 0.001) and be treated in small bed sized hospitals (25.3 % vs 21.1 %, *p* = 0.002, Table 1). In terms of comorbidities, non-acute care transfers had higher rates of chronic obstructive pulmonary disease (19.6 % vs 19.0 %, p = 0.002, Table 1), but decreased rates of coronary artery disease, chronic kidney disease, hypertension, obesity, prior myocardial infarction and type 2 diabetes mellitus (Table 1).

**Table 1:** Baseline characteristics of non-acute care transfers and regular hospital admissions before propensity score matching.

	Non-acute care transfers	Regular hospital admissions	*p*-Value
n	35,010	951,189	
Age (mean)	77.2	71.2	<0.001
Sex (%)			<0.001
Male	15,229 (43.5)	496,521 (52.2)	
Female	19,781 (56.5)	454,668 (47.8)	
Race (%)			<0.001
Asian	350 (1.0)	19,975 (2.1)	
Black	4341 (12.4)	176,921 (18.6)	
Hispanic	1225 (3.5)	72,290 (7.6)	
Native American	210 (0.6)	4756 (0.5)	
Other	630 (1.8)	20,926 (2.2)	
White	28,253 (80.7)	656,320 (69.0)	
Median household income by quartile (%)			<0.001
Quartile 1 ($1–47,999)	9663 (27.6)	313,892 (33.0)	
Quartile 2 ($48,000-60,999)	9593 (27.4)	252,065 (26.5)	
Quartile 3 ($61,000-81,999)	8122 (23.2)	220,676 (23.2)	
Quartile 4 ($82,000+)	7632 (21.8)	165,507 (17.4)	
APR-DRG Mortality Score^a^ (%)			<0.001
Score = 0–1 (Minor)	1610 (4.6)	88,461 (9.3)	
Score = 2 (Moderate)	7947 (22.7)	298,673 (31.4)	
Score = 3 (Major)	15,755 (45.0)	406,158 (42.7)	
Score = 4 (Extreme)	9698 (27.7)	156,946 (16.5)	
Payer status (%)			<0.001
Medicare	29,058 (83.0)	686,758 (72.2)	
Medicaid	1926 (5.5)	105,582 (11.1)	
Private Insurance	2766 (7.9)	114,143 (12.0)	
Self-Pay	420 (1.2)	26,633 (2.8)	
No charge	0 (0)	1902 (0.2)	
Other	805 (2.3)	16,170 (1.7)	
Hospital region (%)			<0.001
Northeast	9558 (27.3)	184,531 (19.4)	
Midwest or north central	10,888 (31.1)	214,018 (22.5)	
South	10,678 (30.5)	374,768 (39.4)	
West	3886 (11.1)	178,824 (18.8)	
Hospital location & teaching status (%)			0.001
Rural	5602 (16.0)	120,801 (12.7)	
Urban non-teaching	8052 (23.0)	258,723 (27.2)	
Urban teaching	21,391 (61.1)	571,665 (60.1)	
Hospital bed size (%)			0.002
Small	8858 (25.3)	200,701 (21.1)	
Medium	9663 (27.6)	280,601 (29.5)	
Large	16,490 (47.1)	470,839 (49.5)	
Comorbidities (%)			
Coronary artery disease	13,560 (38.7)	399,315 (42.0)	<0.001
Chronic kidney disease	1330 (3.8)	44,820 (4.7)	<0.001
Chronic obstructive pulmonary disease	6870 (19.6)	180,345 (19.0)	0.002
Hypertension	9075 (25.9)	305,260 (32.1)	<0.001
Obesity	5090 (14.5)	171,785 (18.1)	<0.001
Previous myocardial infarction	3160 (9.0)	104,009 (10.9)	<0.001
Type 2 diabetes mellitus	7615 (21.8)	238,635 (25.1)	<0.001

a APR-DRG scores are calculated from discharge billing codes and based on discharge diagnosis, pre-existing medical conditions and age.

**Fig. 1:**
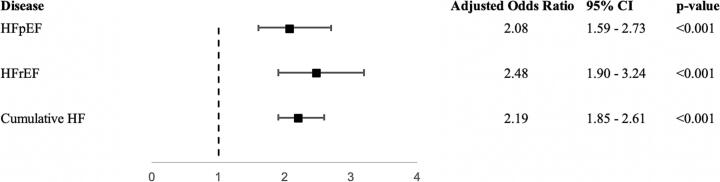
Adjusted odds ratios comparing disease mortality in non-acute care transfers and regular admissions. Adjusted odds ratios calculated by multivariable logistic regression, adjusted for age, race, sex and comorbidities.

After nearest neighbor propensity-score matching, 6689 patients were in each cohort. Both cohorts had similar mean age, sex, median household income, hospital bed size, hospital region and hospital location distributions (Table 2). When adjusted for age, race, sex and comorbidities, non-acute care transfers with congestive heart failure had 2.20 times higher odds of suffering in-hospital mortality compared to regular, non-transferred admissions (aOR 2.20, 95 % CI: 1.85–2.61; *p* < 0.001, Fig. 1). When separated by diagnosis, non-acute care transfers still had a higher likelihood of mortality as well. Non-acute care transfers with heart failure with preserved ejection fraction (HFpEF) had 2.08 times higher odds of suffering in-hospital mortality compared to regular admissions (aOR 2.08, 95 % CI: 1.59–2.73; *p* < 0.001, Fig. 1). Non-acute care transfers with heart failure with reduced ejection fraction (HFrEF) had 2.48 times higher odds of suffering in-hospital mortality compared to regular admissions (aOR 2.48, 95 % CI: 1.90–3.24; *p* < 0.001, Fig. 1).

**Table 2:** Baseline characteristics of non-acute care transfers and regular hospital admissions after propensity score matching.

	Non-acute care transfers	Regular hospital admissions	p-Value
n	6689	6689	
Age (mean)	77.3	77.3	0.88
Sex (%)			0.70
Male	2901 (43.4)	2924 (43.7)	
Female	3788 (56.6)	3765 (56.3)	
Race (%)			<0.001
Asian	64 (1.0)	73 (1.1)	
Black	834 (12.5)	791 (11.8)	
Hispanic	232 (3.5)	315 (4.7)	
Native American	36 (0.5)	17 (0.3)	
Other	118 (1.8)	69 (1.0)	
White	5405 (80.8)	5424 (81.1)	
Median household income by quartile (%)			0.11
Quartile 1 ($1–47,999)	1830 (27.7)	1846 (27.9)	
Quartile 2 ($48,000-60,999)	1792 (27.2)	1732 (26.2)	
Quartile 3 ($61,000-81,999)	1521 (23.1)	1668 (25.3)	
Quartile 4 ($82,000+)	1454 (22.0)	1361 (20.6)	
APR-DRG Mortality Score^a^ (%)			
Score = 0–1 (Minor)	302 (4.5)	399 (6.0)	
Score = 2 (Moderate)	1531 (22.9)	1919 (28.7)	
Score = 3 (Major)	3015 (45.1)	3087 (46.2)	
Score = 4 (Extreme)	1841 (27.5)	1284 (19.2)	
Payer status (%)			0.002
Medicare	5561 (83.1)	5606 (83.8)	
Medicaid	371 (5.6)	369 (5.5)	
Private insurance	517 (7.7)	540 (8.1)	
Self-pay	83 (1.2)	87 (1.3)	
No charge	3 (0.1)	6 (0.1)	
Other	154 (2.3)	81 (1.2)	
Hospital region (%)			0.53
Northeast	1877 (28.1)	1916 (28.6)	
Midwest or north central	2009 (30.0)	1872 (28.0)	
South	2066 (30.9)	2120 (31.7)	
West	737 (11.0)	781 (11.7)	
Hospital location & teaching status (%)			0.31
Rural	1036 (15.5)	952 (14.2)	
Urban non-teaching	1558 (23.3)	1676 (25.1)	
Urban teaching	4095 (61.2)	4061 (60.7)	
Hospital bed size (%)			0.38
Small	1698 (25.4)	1582 (23.7)	
Medium	1847 (27.6)	1947 (29.1)	
Large	3144 (47.0)	3160 (47.2)	
Comorbidities (%)			
Coronary artery disease	2573 (38.5)	2523 (37.7)	0.37
Chronic kidney disease	254 (3.8)	221 (3.3)	0.12
Chronic obstructive pulmonary disease	1313 (19.6)	1221 (18.3)	0.04
Hypertension	1751 (9.1)	1769 (26.4)	0.73
Obesity	971 (26.2)	864 (12.9)	0.01
Previous myocardial infarction	599 (9.0)	542 (8.1)	0.08
Type 2 diabetes mellitus	1456 (21.8)	1444 (21.6)	0.8

a APR-DRG scores are calculated from discharge billing codes and based on discharge diagnosis, pre-existing medical conditions and age.

Non-acute care transfers were more likely to have acute kidney injury (OR 1.11, 95 % CI: 1.05–1.17; p < 0.001), atrial fibrillation (OR 1.12, 95 % CI: 1.05–1.20; p < 0.001) and cardiogenic shock (OR 1.25, 95 % CI: 1.07–1.46; *p* = 0.004). Non-acute care transfers were less likely to have supraventricular tachycardia (OR 0.80, 95 % CI: 0.67–0.97; *p* = 0.02) and undergo percutaneous coronary intervention (OR 0.73, 95 % CI: 0.52–1.03; *p* = 0.07). There was no significant difference between our two cohorts in terms of atrial flutter (OR 0.69, 95 % CI: 0.41–1.16; *p* = 0.16), ventricular fibrillation (OR 1.07, 95 % CI: 0.70–1.63; *p* = 0.77) and ventricular tachycardia (OR 0.92, 95 % CI: 0.83–1.03; p = 0.16). Non-acute care transfers also had an average length of stay of 6.2 days (Median 5 days, IQR 3–7 days), compared to an average length of stay of 5.3 days (Median 4 days, IQR 3–6 days) for non-transferred hospital admissions (*p* < 0.001) and tended to have higher total hospital costs ($49,876.62 vs $44,524.96, *p* = 0.03).

**Fig. 2:**
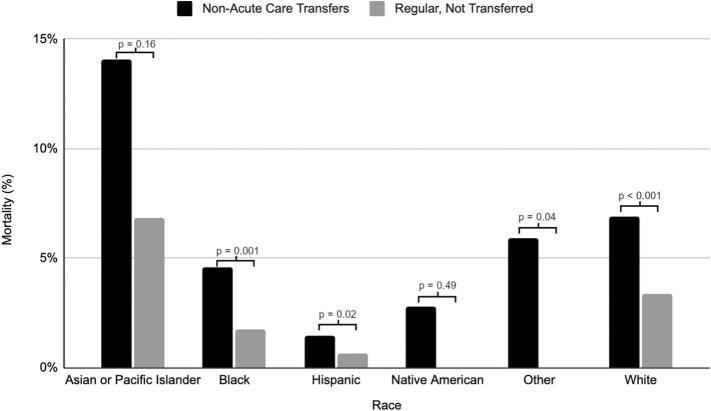
Mortality separated by race.

When separated by race, every race had higher rates of mortality in the non-acute care transfer cohort – Asian (14.1 % vs 6.9 %, *p* = 0.16), Black (4.6 % vs 1.8 %, *p* = 0.001), Hispanic (1.5 % vs 0.6 %, *p* = 0.02), Native American (2.8 % vs 0 %, *p* = 0.49), Other (5.9 % vs 0 %, *p* = 0.04) and White (6.9 % vs 3.4 %, *p* < 0.001, Fig. 2). Asian non-acute care transfers had an average length of stay of 5.6 days (Median 5 days, IQR 3–8 days), compared to an average length of stay of 6.8 days (Median 4 days, IQR 3–6 days) for non-transferred hospital admissions (*p* = 0.27) and had lower total hospital costs ($54,160.82 vs $79,707.86, *p* = 0.06). Black non-acute care transfers had an average length of stay of 7.0 days (Median 5 days, IQR 3–8 days), compared to an average length of stay of 5.4 days (Median 4 days, IQR 2–7 days) for non-transferred hospital admissions (*p* < 0.001) and had higher total hospital costs ($58,434.59 vs $47,374.69, p = 0.06). Hispanic non-acute care transfers had an average length of stay of 6.6 days (Median 5 days, IQR 3–9 days), compared to an average length of stay of 5.5 days (Median 4 days, IQR 3–7 days) for non-transferred hospital admissions (*p* = 0.01) and had higher total hospital costs ($79,693.56 vs $61,410.49, *p* = 0.10). Native American non-acute care transfers had an average length of stay of 6.8 days (Median 5 days, IQR 2–9 days), compared to an average length of stay of 5.4 days (Median 5 days, IQR 3–6 days) for non-transferred hospital admissions (*p* = 0.20) and had higher total hospital costs ($64,696.62 vs $43,911.58, p = 0.20). Other non-acute care transfers had an average length of stay of 8.8 days (Median 5 days, IQR 4–10 days), compared to an average length of stay of 4.9 days (Median 3 days, IQR 2–6 days) for non-transferred hospital admissions (*p* < 0.001) and had higher total hospital costs ($93,046.35 vs $51,419.89, *p* = 0.05). White non-acute care transfers had an average length of stay of 6.0 days (Median 4 days, IQR 3–7 days), compared to an average length of stay of 5.2 days (Median 4 days, IQR 3–6 days) for non-transferred hospital admissions (p < 0.001) and had higher total hospital costs ($46,194.22 vs $41,622.74, *p* = 0.08).

## 4 Discussion

In our nationally representative sample of inpatient hospitalizations across the United States, non-acute care transfers with congestive heart failure had higher odds of suffering in-hospital mortality compared to regular, non-transferred hospital admissions. Even when separated by race, patients transferred from non-acute care centers had higher odds of inpatient mortality. Non-acute care transfers were also more likely to have acute kidney injury, atrial fibrillation and cardiogenic shock. Our findings are highly generalizable given the large, heterogeneous population that was studied.

There are some plausible theories as to why congestive heart failure patients transferred from non-acute care centers are more likely to suffer in-hospital mortality. One possible explanation is that non-acute care facilities, such as urgent care centers and nursing homes, are not as well-equipped with the same resources, (imaging modalities, medications, specialized staff, etc.), as hospitals are for managing acutely ill patients [1], [7], [8]. The lack of these resources can cause delays in diagnosis, leading to adverse outcomes.

The location of a non-acute care center can also impact a patient's outcome. According to the Urgent Care Association's *2018 Benchmarking Report*, only 6.7 % of urgent care centers can be found in rural areas [1], [5]. Furthermore, 138 rural hospitals have closed since 2010 and in February 2019, 673 additional rural hospitals were at risk of closing as well [5], [11]. This additional time it takes for a patient to find a healthcare provider or be transferred to an acute care center from an underserved, rural location can also negatively impact a patient's outcome [12], [13], [14].

Another alternative explanation is that some patients are using non-acute care centers as their primary source of healthcare. In the Urgent Care Association's *2018 Benchmarking Report*, it was reported that 35 % of urgent care patients are unaffiliated with a primary care provider or medical home [15]. While urgent care centers have their own utility within our medical system, it is important for patients to have regular follow-up with a primary care provider for close management of their chronic illnesses.

Additionally, the shortage of physicians and nurses is another contributing factor. A 2021 survey conducted by the American Health Care Association found that 99 % of nursing homes and 96 % of assisted living facilities endorsed facing staffing shortages [16]. Unfortunately, this issue does not seem to be rectified soon. The Association of American Medical Colleges predicts a shortage of between 37,800 and 124,000 physicians in the United States by 2034 [17]. This high patient-to-provider ratio allows for less time to be spent with each patient, increasing the chances for medical errors to occur. Furthermore, along with this shortage of physicians, our older population continues to grow. The Population Reference Bureau predicts that by 2060, there will be 95 million Americans ages 65 and older, nearly double from 2018 [18]. As our population's average life expectancy increases, chronic diseases will need closer follow-up and management. We must continue to find innovative solutions to address this shortage of healthcare providers.

Given that non-acute care centers are also a staple of medical systems in other countries, this study is highly generalizable on a global scale as well. For instance, the *Market Research Report* from March 2018 projected that the global urgent care center market will grow from 19.2 billion dollars in 2018 to 25.9 billion dollars in 2023 [19]. While North America accounts for most of this development, Europe and Asia are also areas of growth as well. Furthermore, based on a United Nations study from 2017, Asia is expected to see a twofold increase in the number of individuals over the age of 60 from 549 million in 2017 to 1.3 billion in 2050 [20]. Thus, integrated healthcare systems, which utilize non-acute care centers, are becoming increasingly popular [21]. This study sheds light at both a national and international level on the importance of non-acute care centers and how vital they are to the health and well-being of elderly populations.

Our findings illustrate that non-acute care transfers are a vulnerable population that require additional medical support in the acute care setting. This study highlights the need for further health policy discussion, specifically pertaining to the design and structure of these non-acute care centers. Furthermore, an increased emphasis on medical resource reallocation to help improve accessibility and the quality of healthcare received by this vulnerable population should also be considered.

Our study has some limitations. Firstly, this was a retrospective cohort study done by using an administrative database, which limits the uniformity of data collection and has the potential for misclassification. When using a database such as NIS, there is an inherent risk for miscoding and under coding as it was not possible for us to determine the diagnostic criteria used at each hospital. Data such as the left ventricular ejection fraction, medications administered, readmission rate and outcomes after discharge was also not available. Furthermore, out of the 35,010 non-acute care transfer patients in our dataset, only 6689 (19.1 %) were included in our final analysis. This causes a selection bias and potentially affects the generalizability of our results as these patients were chosen from the calculated propensity scores. Secondly, in the pre-matched data in Table 1, the non-acute care transfers tended to have higher APR-DRG Mortality scores. Thus, it is possible that the non-acute care centers worked as triage centers and selectively transferred sicker patients, while taking care of less sick patients locally. This also creates the potential for selection bias. Propensity score matching helped overcome this possible issue. Further research should be conducted to better understand why non-acute care transfers are more likely to suffer in-hospital mortality and what changes can be made to address this issue moving forward.

## 5 Conclusion

Among patients with congestive heart failure, non-acute care transfers had higher odds of suffering in-hospital death compared to regularly admitted, non-transferred patients. Non-acute care transfers with congestive heart failure are a high-risk population that requires close inpatient monitoring. Further discussion focusing on better supporting this vulnerable cohort in the acute care setting and improving the design and structure of these non-acute care centers should be a point of emphasis moving forward.

## Funding

This research received no grants from any funding agency in the public, commercial or not-for-profit sectors.

## Ethical statement

No publishing ethics, as per the ELSEVIER website, have been violated.

## Declaration of competing interest

The authors have no relevant financial or non-financial interests to disclose.

## Appendix A Congestive heart failure ICD-10 codes

I50.20, I50.21, I50.22, I50.23, I50.30, I50.31, I50.32, I50.33, I50.40, I50.41, I50.42, I50.43.
